# Development of Embroidery-Type Sensor Capable of Detecting Respiration Using the Capacitive Method

**DOI:** 10.3390/polym15030503

**Published:** 2023-01-18

**Authors:** Ji-Seon Kim, TranThuyNga Truong, Jooyong Kim

**Affiliations:** Department of Smart Wearables Engineering, Soongsil University, Seoul 06978, Republic of Korea

**Keywords:** capacitive pressure sensor, respiratory sensor, electro-textile, wearable sensor, embroidery-type sensor

## Abstract

This study presents a respiration sensor that is dependent on a parallel capacitor, including connection lines and electrodes embroidered on textiles. First, characterizations of the respiration capacitor using a silver thread, including a combination of porous Eco-flex simulating air in the lungs due to respiration, were evaluated using an LCR meter. Second, the effects of air gaps on the detection of respiration motions according to the change in electrode distance under pressure were presented. The data values were measured from 1 to 300 kHz using an LCR meter and dielectric test fixture. Third, actual breathing was examined in four patterns: normal breathing, deep breathing, hyperventilation, and apnea. The test was performed after fabricating a clothing-type breathing sensor. Finally, the change in capacitance for actual respiration was determined by wearing a clothing-type respiration sensor based on the data collected. The effectiveness of the respiration sensor was demonstrated by measuring it to discern all waveforms, cycles, and ranges associated with the breathing pattern.

## 1. Introduction

In recent years, monitoring physiological data using wearable health sensors has become a fundamental feature of health and well-being applications. Some recent studies have used humidity and temperature created from breathing as a signal to recognize human breathing rate [1,2,3]. These studies take the initiative to utilize nonwoven face masks using relative humidity changes to measure human respiration. More importantly, information on respiration helps determine health. For example, wearable respiration sensors can detect apnea episodes during sudden infant death syndrome and sleep apnea and track a person’s physiological state during anesthesia, surgery, trauma, cardiac arrest, and acute disease [4]. Moreover, the breathing rate can be utilized to accurately assess respiration to detect dynamic health conditions, which involves monitoring rising and falling breaths, and other examples include tracking the physiological condition and performance of firefighters or soldiers.

Several commercially available contact spirometers are regularly used as the standard for respiratory rate monitors to detect respiration. Depending on the context, such different positions can be used to place sensors on the body, such as a mask on the mouth [3,5] and tight-worn chest belts [6]. These studies detected fluctuations in respiratory airflow velocity or volume. For instance, a capacitive flow sensor has been reported, where capacitance variations in the sensor plates were triggered by detecting the airflow-generated changes. These sensors detect airflow fluctuations to monitor respiration [7]. However, because it does cover the mouth, a belt-type strain sensor utilizing piezoresistive fabric has been investigated [8]. Based on the sensing principle, the inner garment surrounding the body invariably creates stress. This stress severely burdens people with poor health [9]. Moreover, in terms of choosing conductive yarns [10], they selected nickel, known as stainless steel, as the core fiber twisted together with terylene yarn to develop conductive fabrics. Alternatively, in a separate study, the sensor was attached to nonconductive materials, such as T-shirts, to quantify breathing. However, these designs are difficult to connect to clothing or belts, and the attachment component is prone to deterioration owing to body sweat or external environmental variables.

Consequently, a number of sensors that can be used to monitor physical activity, such as breathing rate, have been proposed [11,12]. However, these sensors are often extensive and obtrusive to the user’s daily activities and must be installed by trained persons [13]. Besides, contact with electronic equipment over an extended period can cause skin irritation and discomfort. Furthermore, the sensitivity of electronic respiratory sensors to electromagnetic interference limits their use in high-electromagnetic environments, such as in magnetic resonance imaging (MRI) applications [14,15].

This study attempts to detect breathing by developing a light and simple electrode that can be worn in a comfortable clothing form. The electrodes (circuit and connector) were composed of a conductive thread and were created by embroidery. The capacitance was determined using the capacitance principle, with two electrodes facing the center of the human body. The difference in air permittivity owing to respiration is the difference in the change in the distance between the electrodes. The electrodes were evaluated by adjusting the embroidery thread type, angle, and density factors. A comparative experiment for air permittivity was performed by varying the mass of air in Eco-flex. A sample experiment was conducted using an LCR meter, which was then made into a garment type and directly worn, with respiration being measured.

## 2. Materials and Methods

### 2.1. Electrodes and Sewing Process

**Figure 1:**
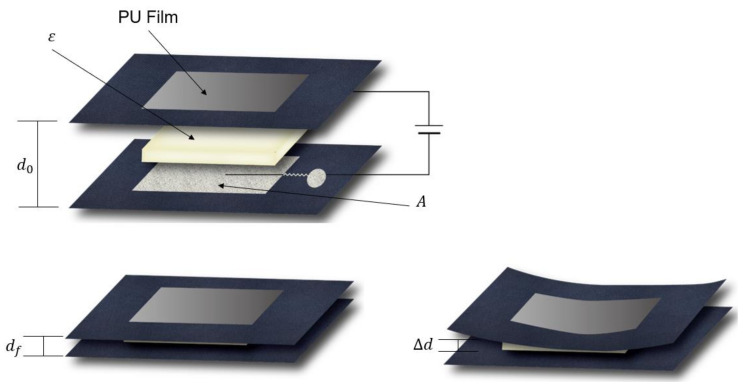
Principle of capacitance measurement of fabricated electrodes.

The polyester fabric was used as a substrate for flexible pressure sensors owing to its flexibility and suitability for human body curves. Moreover, by combining silver threads, known as conductive materials, with textile-like polyesters, fabricated samples will have a hybrid conductive structure, which promotes the capacity to produce sensors with larger regions, which is generally unachievable with other materials. Each detector electrode consisted of two parts: a nonconductive polyester covered by a polyurethane (PU) film on the top and a conductive layer embedded by a machine (Brother PR670E, Cranleigh, UK) using a silver-coated conductive yarn (AMANN silver-tech, Houston, TX, USA) at the bottom (shown in Figure 1).

**Figure 2:**
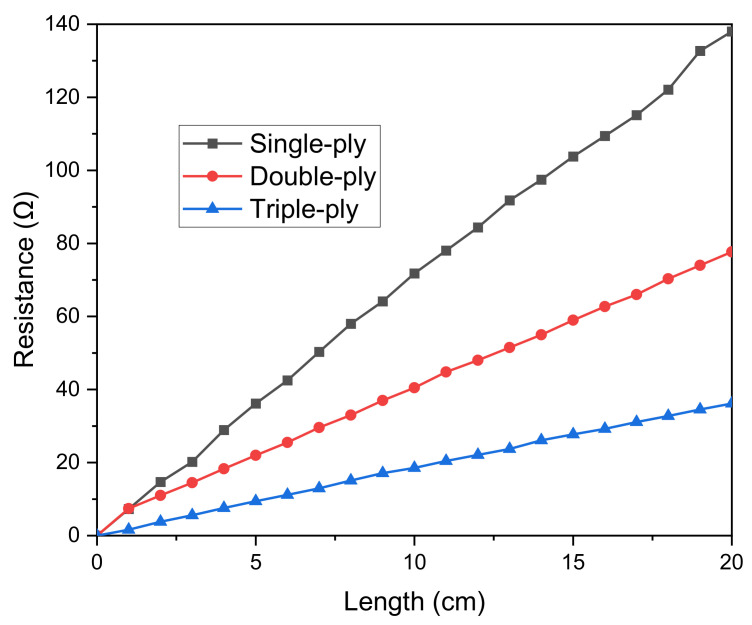
Effect of single-ply, double-ply, and triple-ply thread length on the resistance value.

Silver-coated outer nylon cores used in this study are twisted 34 filaments to create a single conductive yarn. We selected this silver-tech conductive yarn because of its high conductivity and low corrosion resistance, which is ideal for conductive surfaces [16], particularly for sewing or embroidery [17]. The electrical resistance of each ply of the yarn, as determined using an LCR meter, is depicted in Figure 2. As the length increases, the resistance increases and decreases as the number of plies increases [18]. This indicates that the conductive wire of the electrode has a low resistance, making it an appropriate material for use as a capacitance sensor. Additionally, since there was no cellular damage in the cytotoxicity test carried out in accordance with the Biological evaluation of medical devices standard (DIN EN ISO 10993-5), it can be used in daily life without harming the human body. The embroidery Brother PR670E has a straightforward production process, which allows for achieving high geometrical accuracy in unconstrained placement and is easily implemented in various shapes. Resultantly, a conductive silver thread and an embroidery machine were used in this study to embroider sensor electrodes directly to make them easy and sturdy. Furthermore, the connection terminal portion for measuring the capacitance and the circuit connecting the connection terminals were fabricated by embroidering.

**Figure 3:**
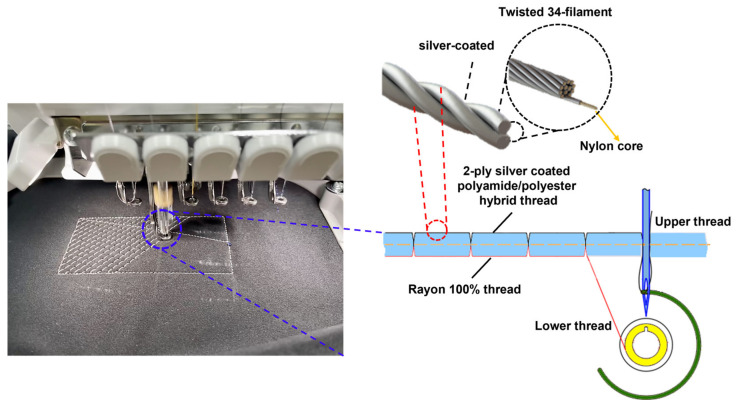
Embroidery process.

During the weaving process, thread strength reduction is an undesirable effect. This is because, at a high velocity, the surface contact of the silver layer builds the string pressure, which prompts the breaking strength of the conductive string during the interaction. Accordingly, to sew conductive layers on both sides, the speed of the embroidery process must be reduced, thereby increasing the assembly cost and time utilization. In this study, the conductive string was utilized exclusively for one side, the base or bottom side, and the rayon string on the top side, as shown in Figure 3. Rayon yarn is the most commonly used thread in the weaving industry. It has an attractive sheen and is moderately priced. The average breaking force and elongation graph presented in [18] indicates that rayon thread is stronger than single-ply thread and weaker than double-ply thread. This is because the elongation of rayon is the shortest, and the single-ply string is the longest, leading to rayon threads stretching more petite, which prevents them from pushing further, retaining the original shape of the capacitor sensor. Thus, the fabricated sensor can deal with the right way with less tensions and increasing stabilization. The connection circuit design was stitched with a silver-coated conductive yarn in a zigzag pattern (Table 1).

### 2.2. Measurement Principle

The electrodes were fabricated using the capacitance approach. As illustrated in Figure 1, a dielectric layer was placed between the two electrodes. In this case, the change in the dielectric constant due to external pressure is similar to the simulation of the presence of air and the absence of air that occurs in human lungs when the person inhales and exhales. Theoretically, the capacitance of a parallel-plate capacitor is given by formula (1):

(1) $$C=\frac{\varepsilon_{r}\varepsilon_{0}A}{d},$$

where $\varepsilon_{r}$ represents the dielectric constant of the material, $\varepsilon_{0}$ indicates the vacuum permittivity, *A* denotes the effective area of the upper and lower plates, and *d* represents the thickness or the spacing between the two electrodes.

The capacitance change in the proposed sensor, known as the distance between the electrodes (d), was monitored. In this approach, the thickness or dielectric layer changes under external force, simultaneously leading to variation in the capacitance of the sensor. Due to dependence on the parameters *A* and *d* in Formula (1), changing the area or thickness affects the pressure sensitivity. Therefore, when the proposed sensor is applied to the body, three main problems affect the performance of our sensor. The first is the dielectric properties of the skin. Under the activity of human breathing, the skin layer will come into contact with the electrode layers. Depending on the intensity of inhalation, this contact will create a slight or intense pressure between the electrode layers and the skin. Initially, we expected this to be the main problem affecting our sensor to detect human breathing. Under pressure, the dielectric constant of the skin layer will increase [19], leading to an increase in capacitance. However, the experimental results showed a different context than predicted. This discussion will be gone further in the results section. The second is the gap between two electrodes. When breathing in, the distance between the electrodes will increase and decrease when breathing out. Additionally, final is the amount of air in the lungs due to respiration. Our experiments were performed with two goals. First, whether there is an effect of the electrode spacing on the embroidery sensor. If so, this effect is large or small. Second, test the proposed sensors’ sensitivity with and without air inside the dielectric layer, simulating the perspective air in the lungs due to respiration.

### 2.3. Dielectric Layer

Eco-flex (Smooth-on Inc., Macungie, PA, USA) was employed to create an ideal dielectric environment with and without air, which was used in the experiment to detect respiration motion. Eco-flex was chosen as the dielectric due to its low viscosity, making it simple to mix with other materials and causing no surface-level phenomena [20]. It is also safe for the human body and is easy to create in complex shapes. An Eco-flex was employed to measure the change in permittivity ($\varepsilon_{r}$).

**Figure 4:**
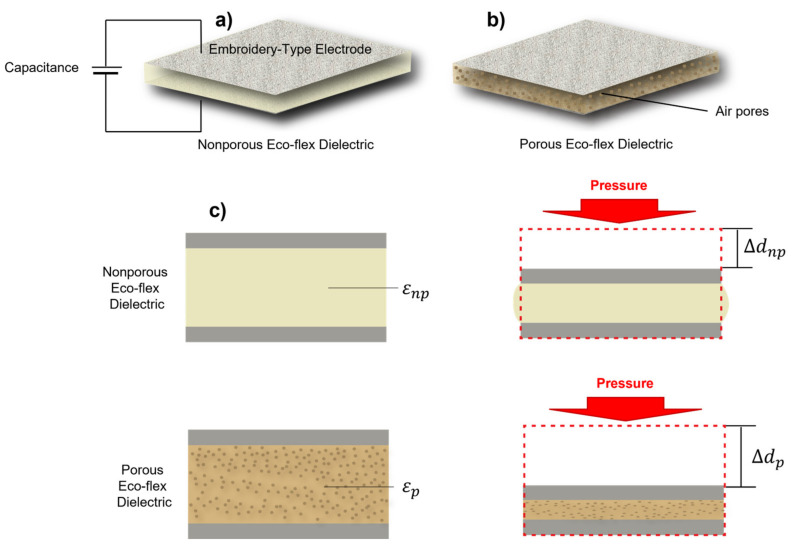
Pressure sensing mechanisms of the capacitive respiratory sensor using a nonporous eco-flex dielectric and porous Eco-flex dielectric. (**a**) Diagram showing the structural deformation of a capacitive respiratory sensor utilizing an Eco-flex dielectric layer that is nonporous. Porous is shown in (**b**). Schematic of the structural deformation of the capacitive respiratory sensor under compressive loading utilizing a nonporous, porous Eco-flex dielectric layer (**c**).

**Figure 5:**
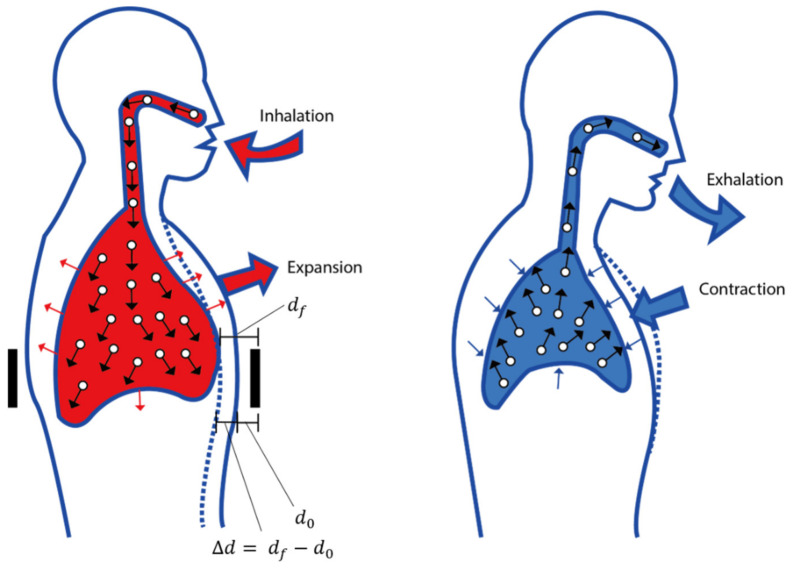
Changes in the abdomen size and the amount of air in the lungs due to respiration.

To study the effect of various amounts of air in the Eco-flex samples, an LCR meter (E4980AL) was set up with 201 points ranging from a frequency of 10 to 300 kHz. The measurements under 10 kHz were unstable; therefore, all experiments were performed over a frequency range of 10 to 300 kHz. Furthermore, the capacitance change values under pressure were calculated when the same was applied with a pressure tester (Dacell Co., Seoul, Republic of Korea) during periods of 10, 45, and 65 kHz. In this study, we assumed that the human body is likely a dielectric layer, electrodes were placed on the front and rear of the body’s inner thigh, and the amount of air ($\varepsilon_{air}$) in the lungs varies when inhaled and exhaled (Figure 4). Moreover, the capacitance changes owing to the difference at the moment ($d_{f}$-$d_{0})$, according to the principle of capacitance, as depicted in Figure 5.

In this phenomenon, the nonporous Eco-flex ($\varepsilon_{np}$) dielectric constant is almost unchanged with pressure, so the variations of its capacitance sensor are dependent on the changes in the distance between the two electrodes ($\Delta d_{np})$, as illustrated in Figure 5. In contrast, the dielectric constant and the distance between the electrodes ($\Delta d_{p})$ vary under pressure in the case of a porous Eco-flex ($\varepsilon_{p}$), in which an air layer is produced by sugar. Additionally, even under the same pressure, the capacitance of the porous Eco-flex changes significantly, indicating that the shift in capacitance is substantial [20]. The effective dielectric constant of porous Eco-flex ($\varepsilon_{r}$), which may be explained by Formula (2) [21], increases when the pores are subjected to external pressure [22]:

(2) $$\varepsilon_{r}=\varepsilon_{air}V_{air}+\varepsilon_{Eco-flex}V_{Eco-flex},$$

where $v_{air}$ indicates the volume fraction of air and $V_{Eco-flex}$ is for the volume fraction of the immaculate Eco-flex, where $\varepsilon_{air}=1$ and $\varepsilon_{Eco-flex}=2.8$ [23]. The compression causes the pores of the dielectric layer to close gradually, which lowers the volume percent of air and increases the volume fraction of Eco-flex. The greater dielectric constant of the silicone elastomer ($\varepsilon_{Eco-flex}=2.8$) replaced the lower dielectric constant of the pores ($\varepsilon_{air}=1$), increasing the effective dielectric constant of the porous Eco-flex composite [20,24]. The sensitivity of the capacitance sensor can be increased by adding air gaps to this porous elastomer owing to increased deformability.

**Figure 6:**
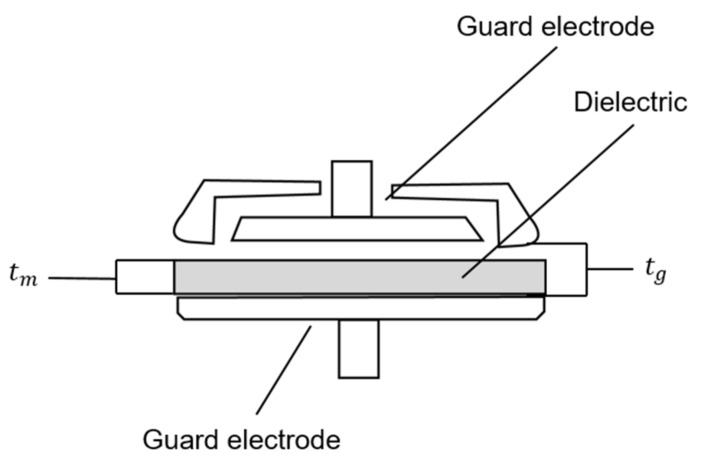
Noncontacting electrode method.

To calculate the permittivity of the Eco-flex samples, multilayer ceramic capacitors (ML-CCs) were used to measure and quantify the dielectric constant as a function of the dielectric change. The 16451 B dielectric test fixture from Keysight was utilized to accurately evaluate the dielectric constant of the Eco-flex samples with and without an air gap. A noncontact approach was used to perform the measurements. The dielectric to be measured was placed at a lower position among the guard electrodes, above and below. In this approach, the upper guard electrode was measured at a particular distance. The thickness of the dielectric to be measured is denoted as $t_{m}$, and the gap formed at this time is denoted as $t_{g}$. To obtain the dielectric constant, two capacitance values are required. First, $C_{s1}$ denotes the capacitance value measured without a known dielectric material under test (MUT), and $C_{s2}$ denotes the capacitance after the MUT has been placed on the guard electrode. The formula for applying this principle can be found in Formula (3), given by the Keysight manual. Figure 6 illustrates the experience measurements.

(3) $$\varepsilon_{r}=\frac{1}{1-\left(1-\frac{C_{s}1}{C_{s}2}\right)\times \frac{t_{g}}{t_{m}}}$$

### 2.4. Fabrication

**Figure 7:**
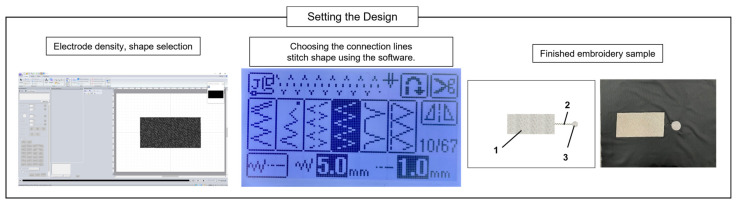
Preprogrammed selected shapes of the electrode, connection line, and connector. (1) embroidery connection lines, (2) embroidery electrode and (3) embroidery connector.

A computerized embroidery machine was employed to fabricate the electrodes of the proposed capacitive sensor and connectors. An automatic embroidery machine program (PE-design) was used to specify the geometry of the electrode and connector, as well as the thread density (Figure 7). The electrode measured 100 mm × 50 mm in width and height, and the connector was 20 mm in diameter. The fabric used for the black jacket consisted of 87% polyester and 13% spandex. Subsequently, the electrodes were sewn directly into the coating on both sheets (front and back). Only the upper sheet was embroidered using a conductive thread, whereas the lower sheet was embellished with a regular embroidery thread. The electrodes were placed facing one another. Accordingly, only the shape of the electrodes was invisible from the outside. More specifically, the outer surface was a rayon thread covered with a layer of a PU film. A PU film was added to create a shield that protected the sensor from environmental disturbances. Figure 1 shows the structure of the manufactured layer. The Eco-flex polymer behaves similar to a human chest. Electrodes fabricated by the embroidery machine have a density of 6 lines/mm, which is called high density. In the experiment, when using a low density for fabrication, the distance between the threads on the electrode surface was widened, reducing the stability of the capacitive sensor. Therefore, for more efficient measurements, a dense density was chosen. As the connectors (number (3) described in Figure 7) need to connect from the electrodes to the device measurements, we employed the default configuration of the software, medium density (4.5 line/mm), which is appropriate. A zigzag pattern (width of 5 mm, height of 1 mm), supplied by an electric sewing machine, was designed to connect line components connecting the electrodes and connectors. In terms of tension and contraction of the fabric due to movement, the zigzag pattern outperformed the straight design in terms of strength and stability (Table 1). The specifications of the respiratory sensor, shown in Figure 7, are listed in Table 2.

**Figure 8:**
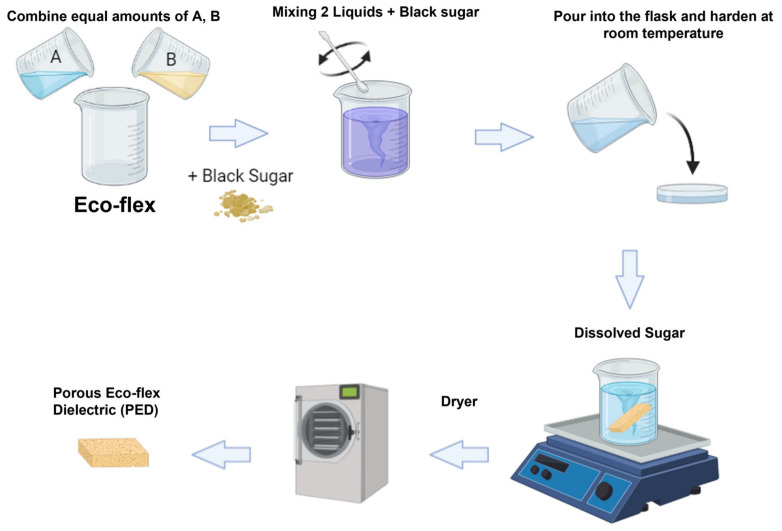
The fabrication process of PED.

**Figure 9:**
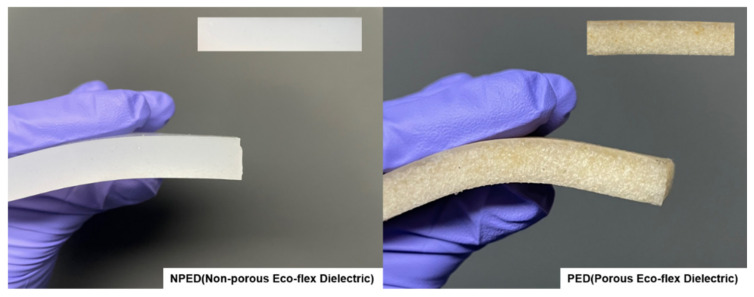
The appearance of manufactured NPED and PED.

The microporous dielectric layer or pressure-sensing layer was fabricated using the process shown in Figure 8. The Eco-flex solution was obtained by mixing a base (Part A) and a cured agent (Part B) with a volume–weight ratio of 1:1. Following this process, granulated brown sugar was dispersed in the Eco-flex solution. The solution was stirred at 120 rpm for 15 min to help segregate granulated brown sugar and evenly distribute it in the composite. The mixture was cured at room temperature (30 °C) for 3 h. To form the shape of the dielectric films, we used a 3D mold with the length, width, and height of the electrodes of the same size (100 mm × 50 mm), as shown in Figure 9. Following the curing time, the mixer was removed from the 3D mold, and the sugar was dissolved in boiler water under magnetic stirring at 200 rpm for at least 24 h. Porous Eco-flex dielectric (PED) is the sample with air due to the formation of pores. In contrast, nonporous Eco-flex dielectric (NPED) is the sample in which no pores were formed because sugar was not utilized (NPED).

**Figure 10:**
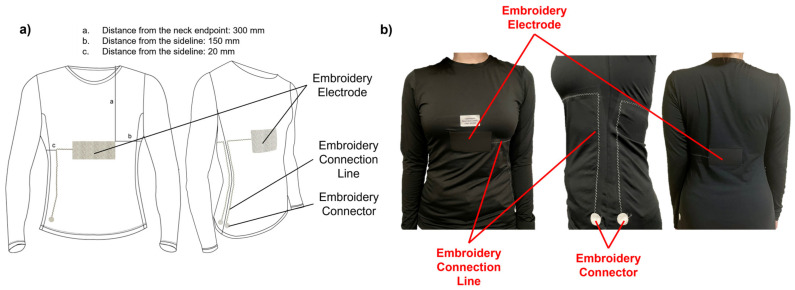
A respiratory sensor in the shape of a finished garment: (**a**) schematic; (**b**) actual photo.

A T-shirt-style respiratory sensor measurement garment was created for the final wear test (Figure 10). The T-shirt, composed of 87% polyester and 13% spandex, was chosen as the product with good activity and elasticity (Sumnfit, Seoul, Republic of Korea). The exact size of the experimental sample (100 × 50 mm) was created to be applied to the abdomen. To minimize any inconvenience when connecting to the device while wearing clothing, the connector section that transmitted the output value was placed near the bottom of the electrode, which was chosen because it had the most significant change during respiration. The electrodes and connections were linked together using a conductor from a circuit schematic. The electrodes and connectors were attached to the interior of the jacket. Since a measurement error could arise as the circuit made contact with the body at this point, the circuit was covered with a polyurethane (PU) film. A schematic of the prototype is shown in Figure 10.

Table 3 includes all materials and methods used in this work together with their purpose.

## 3. Results

**Figure 11:**
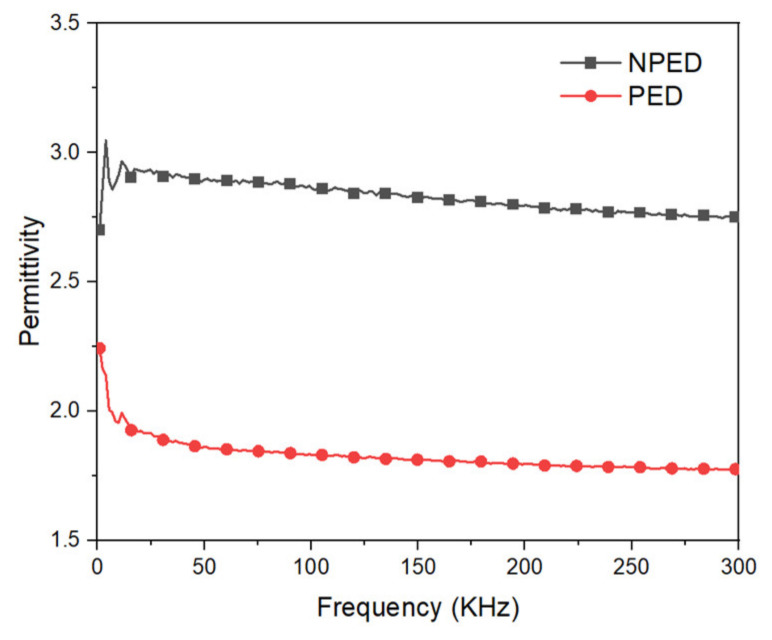
Frequency dispersion of permittivity.

**Figure 12:**
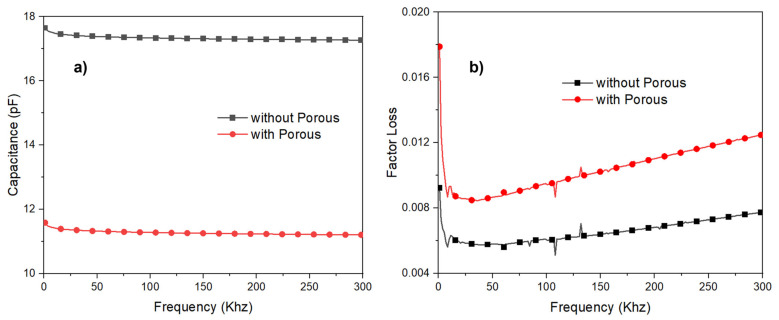
(**a**) Frequency dispersion of capacitance. (**b**) Frequency dispersion of loss tangent.

**Figure 13:**
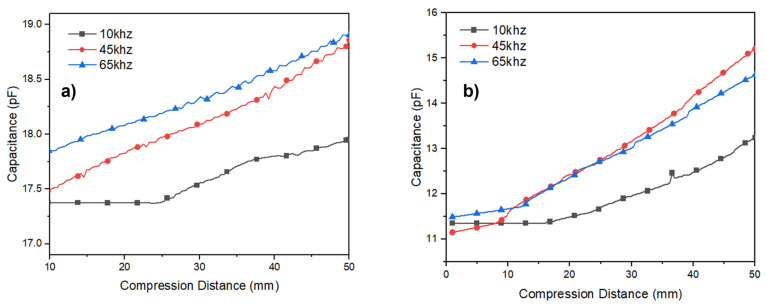
Capacitance changes according to compression distance ($\Delta d_{p})$, (**a**) NPED, (**b**) PED.

The dielectric NPED and PED were calculated using Formula (3). Figure 11 and Figure 12 show the measured frequency values between 1 and 300 kHz. PED indicates that the porous (PED) has a lower permittivity than the nonporous (NPED), as shown in Figure 11, leading to the NPED capacitance sensor observing values between 17.2 and 17.6 pF, while the PED capacitance sensor recorded values between 11.2 and 11.5 pF (Figure 12). NPED and PED displayed an unstable appearance at frequencies below 10 kHz, but the corresponding capacitance values remained steady. Both NPED and PED displayed varying values in the 10 kHz part from the initial measured value in the factor loss of Figure 12, and stable values in the 45 kHz section. It can then be observed that the values increased once more, starting at 50 kHz or greater. Consequently, 45 kHz was chosen as the appropriate frequency for this study because it allowed for the stable measurement of all capacitance sensor values using NPED and PED. Both NPED and PED in the factor loss of Figure 12b exhibited unstable values in the 10 kHz part from the initial measured value; however, the values were stable in the 45 kHz area. It can then be observed that the values increase progressively, starting at 50 to 300 kHz. Resultantly, the frequency chosen for this study was 45 kHz, where the values of the capacitance sensors employing NPED and PED were consistently measured. A comparison of the dielectric constants of the NPED- and PED-based capacitance sensors is presented in Figure 13. The permittivity of NPED was higher, as shown in Figure 12, and it had an unstable value at frequencies of 1 kHz or less, as well as a tendency for a decreasing dielectric constant starting at frequencies of 50 kHz or higher. This result corresponds to that shown in Figure 12.

**Figure 14:**
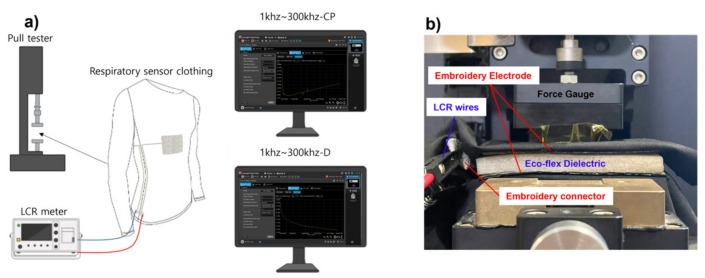
(**a**) Schematic of the universal testing machine. (**b**) Measurement setup for the proposed capacitance respiratory sensor with porous Eco-flex dielectric.

**Figure 15:**
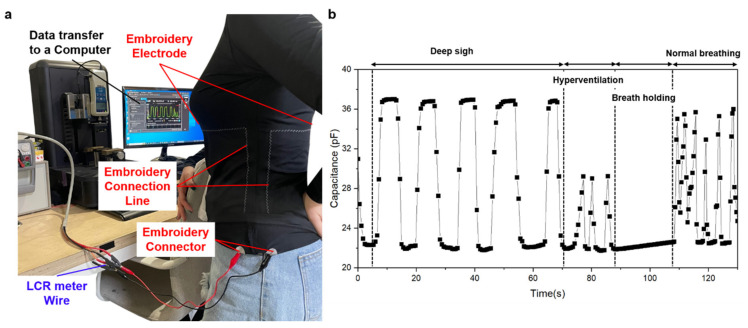
Measurement of respiratory capacitance sensors in clothing. (**a**) Measurement setup for the proposed capacitance respiratory sensor clothing on the body; (**b**) changes in the capacitance according to the breathing pattern after wearing.

The capacitance value according to the dielectric change and the distance between the electrodes at constant pressure was measured using an LCR meter and a pull tester, as shown in Figure 14. The values were determined at three locations with frequencies of 10, 45, and 65 kHz for both the NPED and PED samples. The capacitance value according to the change in the NPED and PED dielectrics is shown in Figure 13, and the distance between the electrodes is conducted according to the compression distance. The compression distance here is ($\Delta d_{p})$, so the greater the pressure, the narrower the distance between the two electrodes, leading to an increase in capacitance. Remember that the capacitance value increases almost linearly and steadily when pressure is applied at a frequency of 45 kHz, as shown in the earlier results of Figure 11 and Figure 12. Additionally, as explained in Figure 4, $\Delta d_{p}$ compresses to a greater degree than $\Delta d_{np}$, and Formula (3) indicates that NPED, a porous dielectric, has better sensitivity. Note that because the sensing capacitor depends on the change permittivity of the MUT (Δε), various capacitances ΔC can be understood by calculating (4):

(4) $$\Delta C=\Delta \varepsilon C_{0},$$

where $C_{0}$ denotes the baseline capacitance or initial capacitance. The capacitance values of interdigitated electrodes are typically low, approximately several femtofarads. Additionally, parasitic capacitances are unwanted elements that can affect the signal-to-noise ratio (SNR) of the readout circuitry system. Therefore, from (3), a higher baseline capacitance is required for more changes in capacitance variance. Furthermore, it is necessary to define that the primary challenge in transferring dielectric changes into pressure is Δε. The higher Δε, the greater the sensitivity that can be achieved. Consequently, the response range of the PED sensor was more expansive. Figure 15 provides additional information on these outcomes. The pressure response time of the proposed sensor was investigated with a rapid recovery time lower than 0.2 s.

Finally, the change in capacitance for actual respiration was determined by wearing a clothing-type respiration sensor based on the data collected thus far. The four breathing patterns measured were the four breathing patterns of deep breathing, hyperventilation, normal breathing, and apnea after donning the T-shirt. As the body volume changes during inhalation and exhalation, the distance between the electrodes also changes, and the capacitance value is calculated in accordance with the change. Owing to the expansion of the abdomen during inhalation, the distance between the electrodes at the front and back of the body increases, leading to a decrease in capacitance at the beginning (can be seen in Figure 15b). Recall that, due to respiration when breathing in, the air in the lungs increases, decreasing the dielectric permittivity. Under pressure, the dielectric constant of the skin layer will increase [19], leading to an increase in capacitance. However, this factor is not as influential as the other two factors, including the distance between two electrodes and the amount of air. In contrast, as the abdomen contracts during exhalation, the position of the electrode moves downward, and the capacitance value increases. The following measurements were taken, as shown in Figure 15a. Using a cable and an LCR wire to link the connector part to the LCR meter, the change in capacitance following the breathing pattern was detected. As shown in Figure 15b, a steady waveform in the vicinity of 22–38 pf was exhibited during deep breathing. As breathing continues slowly, the signal of lingering breathing while holding the breath can also be observed. Rapid breathing was used during hyperventilation, resulting in a waveform with a small breath and a measurement range of 22 pf–30 pf. A waveform with a constant magnitude of approximately 22 pF is visible even during apnea. The dielectric constant of the skin layer generates noise in the upper frequency due to high factor loss (Figure 12b). Therefore, here, we only surveyed the frequency area below 50 kHz and above 10 kHz.

Finally, it exhibits irregular waveforms during normal breathing, as opposed to hyperventilation or apnea, demonstrating a normal breathing rhythm. These findings indicate that the dielectric constant fluctuates as a result of variations in the amount of air in the lungs caused by respiration and that the capacitance fluctuates as a result of variations in the distance between electrodes caused by variations in the volume of the body as a result of respiration. Four types of breathing were considered: regular breathing, hyperventilation, apnea, and deep breathing. As a result of the measurement, it displayed various waveforms based on the breathing pattern and demonstrated usable functionality as a breathing sensor by being able to measure the cycle or pace. Furthermore, owing to its comfort and ease of measurement, it is anticipated that in the future, it will be utilized as a wearable device for real-life or medical purposes if an app for real-time monitoring or a Bluetooth system for wireless measurements is developed.

## 4. Conclusions

In this research, we created a capacitance sensor that can measure breathing, tested its performance by altering the type of Eco-flex, and then created a prototype resembling clothing and assessed its performance through wear testing. A silver-coated conductive yarn was utilized to create flexible, easy, and comfortable electrodes, connection lines, and connectors for sensor fabrication. Using Eco-flex, different volumes of air were created during the sensor evaluation, depending on whether sugar was used during the manufacturing process to alter the dielectric constant. Using an LCR meter, a frequency of 45 kHz, which transmits the most consistent value between 1 and 300 kHz, was chosen, and the measured values of 10 and 65 kHz were compared and measured for each. Additionally, the highest measurement range (NPED: 17–19 pF, PED: 11–15.5 pF) in comparison with the capacitance measured at frequencies of 10 and 65 kHz using a pull tester and LCR meters and the compression distance at constant pressure occurred. Additionally, the change in the capacitance of the Eco-flex PED with pores was greater when the NPED and PED measurement ranges were contrasted. It was discovered that the capacitance measurement range increased with the change in the distance between the electrodes as a result of the change in the amount of air (dielectric constant) in the lungs as a result of respiration and the change in the volume of the body. Upon creating and donning a breathing sensor resembling clothing, we evaluated the actual breathing. The area of the abdomen with the largest volume change was selected for the electrode placement. The connector was positioned at the bottom, connected via an embroidered connection line, and wired to the instrument.

## Figures and Tables

**Table 1:** Form of the stitch changes as the cloth tension changes.

Type	Normal	Stretching
Straight line	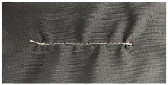	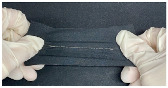
Zigzag line	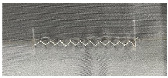	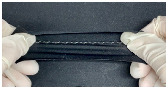

**Table 2:** Specifications of the respiratory sensor.

Parameter	Electrodes (1)	Connection Line (2)	Connectors (3)
Dimension (width × height)	100 × 50 (mm)	25 (mm)	20 × 20 (mm)
Density (line/mm)	6	-	4.5
Shape of the stitch	Running, fill	Zigzag	Running, fill
Length of the stitch (mm)	2, 4	5/1 (width/height)	2, 4
Number of stitches	9526	-	606

**Table 3:** Summary of analyzed materials and test devices used.

Component	Remark	Manufacture	Purpose
Electrodes	Silver-coated outer nylon cores covered by PU	AMANN silver-tech, Augsburg, Germany	Creating the sensing area of the upper and lower plates
Embroidery machine	PR670E	Brother Sewing, Aichi, Japan	Sewing the electrodes and connection wires
Dielectric layer with and without porous	Rubber with and without porous	Eco-flex Smooth-On, Houston, TX, USA	Simulation of air in the lungs due to respiration
Dielectric test fixture	16451B	Keysight Tech, Colorado Springs, USA	Calculate the permittivity of the dielectric samples with and without air
LCR meter	E4980AL	Keysight Tech, Colorado Springs, USA	Analyze the characteristics of respiration sensors
Universal testing machine (UTM)	NA	Dacell Co. Ltd., Seoul, Republic of Korea	Analyze the characteristics of respiration sensors

## Data Availability

Not applicable.
